# Cultivating Disaster Preparedness: Scoping Review of Technology’s Contribution to Situational Awareness and Disaster Mindset in Disaster Medicine

**DOI:** 10.2196/75404

**Published:** 2025-10-10

**Authors:** Amir Khorram-Manesh, Marius Rohde Johannessen, Laurits Rauer Nielsen, Eric Carlström, Lasse Berntzen, Lene Sandberg, Jarle Løwe Sørensen

**Affiliations:** 1 Surgery, Clinical Sciences, University of Gothenburg, Universitetsplatsen 1, Gothenburg, 405 30, Sweden, 46 0317860000, 46 031786497; 2 Department of Business, History and Social Sciences, USN School of Business, University of South-Eastern Norway, Borre, Norway; 3 Management and Administration Emergency and Risk Management, University College Copenhagen, Copenhagen, Denmark; 4 Learning and Leadership for Health Care Professionals, Institute of Health and Care Sciences, University of Gothenburg, Gothenburg, Sweden; 5 Department of Business, Marketing and Law, USN School of Business, University of South-Eastern Norway, Borre, Norway

**Keywords:** disaster education, disaster medicine, disaster mindset, simulation exercises, situational awareness

## Abstract

**Background:**

Disaster medicine education increasingly emphasizes situational awareness and a proactive disaster mindset as crucial competencies for effective response. Situational awareness involves comprehending the disaster environment to make informed decisions under pressure, while a disaster mindset encompasses psychological resilience and effective functioning amid chaos. Integrating technologies into simulation training allows experiential learning that bridges these theoretical concepts with practical application.

**Objective:**

This study aims to investigate the current status of teaching these concepts and the use of technology in fostering situational awareness and a disaster mindset within disaster medicine education by reviewing the existing literature.

**Method:**

This study used a scoping review of scientific studies (2005‐2025), obtained from PubMed, Scopus, and Web of Science databases, complemented by a Google Scholar search. From December 1, 2024, to the end of January 2025, 3 reviewers searched, compiled, reviewed, and selected eligible studies in English, discussing the use of technology in fostering situational awareness and disaster mindset.

**Results:**

Out of 217 initially identified records, 49 studies met the inclusion criteria after a 2-stage screening and full-text review process. Of these, 42 were peer-reviewed scientific articles and 7 were official documents. Approximately 86% (42/49) of the studies addressed situational awareness, while only 2% (1/49) explicitly focused on the concept of disaster mindset. Most of the included studies highlighted the use of immersive technologies such as virtual and augmented reality, geographic information systems, and artificial intelligence–driven tools to enhance real-time information processing and decision-making in disaster education contexts. By strategically incorporating these advanced tools into educational frameworks, the divide between theoretical knowledge and practical application can effectively be bridged, fostering essential experiential learning and developing robust psychological readiness for future challenges.

**Conclusions:**

Simulation training enhances situational awareness and disaster mindset, bridging the gap between theory and practice through experiential learning. The findings from this review highlight current pedagogical approaches and technological applications, identifying gaps and future directions for enhancing disaster medicine education.

## Introduction

The 21st century has experienced a significant transformation in disaster medicine, disaster management, and public health, primarily driven by the rapid advancement of technology. Since the early 2000s, technology has played a pivotal role in shifting these fields from reactive to proactive approaches and from localized to globally interconnected systems [1-3].

Initially, the advent of the internet and mobile communication facilitated faster dissemination of information and coordination during crises [4]. Real-time updates, emergency alerts, and remote communication became essential tools for first responders and public health officials. Geographic information systems (GIS) emerged as a powerful instrument for mapping disaster zones, tracking disease outbreaks, and optimizing resource allocation, providing unprecedented spatial awareness [5]. As technology progressed, its applications expanded. Remote sensing and satellite imagery enabled the early detection of natural hazard-induced disasters, facilitating preemptive evacuations and resource mobilization [6]. The rise of social media platforms introduced new channels for public communication, citizen reporting, and real-time information gathering, although they also presented challenges related to misinformation [7].

The proliferation of mobile health apps and wearable devices has revolutionized public health monitoring and disease surveillance [8]. Real-time data collection, remote patient monitoring, and telehealth services became integral to managing chronic conditions and responding to outbreaks, particularly in resource-limited settings. Furthermore, the development of simulation and virtual reality (VR) technologies transformed training and education in disaster medicine. Immersive simulations allowed health care providers to practice complex procedures and decision-making in realistic, high-stress environments, thereby enhancing preparedness and competency [9].

Artificial intelligence (AI) and machine learning algorithms have emerged as powerful tools for analyzing vast datasets, predicting disease outbreaks, and optimizing disaster response strategies. AI-powered chatbots and online assistants provide instant access to information and support, while robotic systems assist in search and rescue operations [10]. In recent years, the COVID-19 pandemic accelerated the adoption of digital health solutions, underscoring the critical role of technology in pandemic preparedness and response. From contact tracing apps to vaccine distribution platforms, technology has been instrumental in managing the global health crisis [11,12].

While challenges persist, including issues related to data privacy, digital equity, and the ethical use of AI, the transformative role of technology in disaster medicine and public health management is undeniable [13]. By continuing to innovate and integrate new technologies, we can build more resilient communities and enhance global health security.

The current inconsistencies in disaster medicine education stem from the diverse risks, causes, needs, resources, experiences, and expertise inherent to different regions, highlighting a critical need for greater collaboration and a more standardized approach to training. Previous research underscores this requirement, pointing to significant variations in elements of the management systems, including command, control, and communication protocols, alongside notable deficiencies in existing training methodologies [14]. Although disaster medicine education is crucial, it suffers from a lack of global consistency in both content and opportunities, with one major issue being the absence of standardized curricula that adequately address the multifaceted nature of disaster response, including emerging technologies and the complexities of hybrid warfare [14-16]. Insufficient emphasis on practical, hands-on training further hinders effective disaster preparedness, compounded by limited access to advanced simulation and high-fidelity training environments that restrict the ability to provide realistic learning experiences [17]. The high cost of these vital technologies often limits their availability to well-funded institutions, leaving many learners without essential training tools. Moreover, greater interagency collaboration is needed in training programs to ensure all stakeholders, including emergency responders, health care professionals, and government officials, are prepared to coordinate effectively during crises [14-16]. Addressing these shortcomings is essential to improve the effectiveness of disaster medicine education and enhance overall preparedness [14-17].

Globally, the use of simulation exercises in disaster education is recognized. These exercises range from tabletop simulations, which require fewer resources and limited financial support, to live or modular simulations, which demand significantly more resources and funding [18]. In theory, simulation exercises are a potent tool in disaster medicine education, enhancing both learning and practical application [19]. However, they predominantly focus on local and regional group and team activities, while interorganizational, transnational, and global collaboration is still in need of development [19,20]. On the one hand, group dynamics may influence personal beliefs and knowledge, potentially leading to resistance and noncompliance with established guidelines and rules of engagement [21]. On the other hand, routines and technology tend to remain intraorganizational, as well as at local, regional, and national levels [22,23].

These challenges highlight the disconnect between the different management levels, probably stemming from a lack of overall situational awareness (SA) and a disaster mindset (DMS). SA involves the ability to accurately perceive, understand, and respond to elements within a given environment, especially in dynamic and high-stress situations [24]. It encompasses being aware of one’s surroundings and predicting potential changes or threats. A DMS refers to the mental preparedness and resilience necessary to effectively manage and respond to disaster scenarios [25]. It includes maintaining composure, making rapid and confident decisions, and adapting to extreme stress and uncertainty.

The importance of SA and a DMS is increasingly recognized in disaster medicine, necessitating the incorporation of both in current programs and educational initiatives, through lectures, simulations, and field exercises to better prepare responders for the complex psychological and operational challenges they will face. Many programs may use simulations to replicate disaster scenarios, allowing participants to practice SA and decision-making under stress in a controlled environment. In addition, effective disaster response requires teamwork and communication across different disciplines. Training often emphasizes these aspects, which are closely linked to SA and a collaborative mindset [26-32]. Nevertheless, to the best of our knowledge, SA and DMS are not adequately integrated into current educational initiatives in disaster medicine.

This study aims to investigate the status of teaching these concepts and the use of technology in fostering SA and a DMS, both individually and collectively, as well as locally, regionally, and transnationally, by reviewing the existing literature.

## Methods

### Study Method

To comprehensively map the broad field of disaster medicine education, a scoping review was conducted (December 2024 to the end of January 2025), using academic databases (PubMed, Scopus, and Web of Science [WoS]) and Google Scholar in several steps, including search, review, and selection of the literature. To effectively research how technology enhances SA and DMS, a combined search strategy is crucial. Google Scholar excels at identifying a broad spectrum of content, including recent and interdisciplinary work. In contrast, specialized scientific databases offer greater precision through curated, peer-reviewed content, controlled vocabularies, and advanced filters [33]. Using a multidatabase strategy allowed for the capture of a broad research spectrum, providing a robust, unbiased overview of disaster medicine education and ensuring the retrieval of all relevant literature, overcoming the limitations of each when used in isolation. This method also aimed to establish a foundational understanding of the field’s current state by identifying core concepts, theories, and evidence, with particular attention to SA, DMS, and technology’s impact. The review clarified contested definitions, pinpointed critical research gaps, and evaluated the feasibility of a future systematic review [34]. This preliminary assessment serves to guide future research and development in disaster medicine education.

### Research Question

How can current technology help enhance SA and DMS in disaster medicine education?

### Searching Keywords

Disaster AND Technology AND Situational awareness AND Disaster mindset were selected as the final search keywords and deemed highly adequate for investigating the role and impact of technology on SA and DMS in disaster medicine. Disaster is the core subject of this investigation and broadly encompasses the events (natural or human-made) that necessitate disaster medicine and sets the context for all other keywords, avoiding the capture of SA and DSM in nondisaster contexts (eg, routine health care). It ensures that the obtained literature directly addresses the unique challenges and demands of crisis situations. Technology covers a vast array of innovations relevant to disaster medicine, including but not limited to communication technologies (satellite phones, mesh networks, and mobile apps for coordination), information systems (GIS, data analytics, and data processing), remote tools (telemedicine, drones, remote sensors, and wearable devices), and training and simulation (VR and augmented reality [AR]), ensuring the search captures how these tools are being applied and studied within the disaster medicine context. There are several synonyms to use for both SA and DMS, such as perception, comprehension, and cognition for SA, and disaster readiness and resilience for DMS. However, listing all synonyms could potentially lead to information overload, while targeting the core keywords could yield more targeted results.

Educational programs were not included in the main search and were specifically searched through official webpages. The reasons were as follows: (1) adding “educational programs” makes the search even more specific, (2) many studies on technology, SA, and DMS in disaster medicine inherently involve education and training, even if they don’t explicitly use “educational programs” in their keywords or title, (3) educational programs can be very broad, for example, public education and might not involve technology, and (4) their inclusion in every search will constantly result in studies focusing on training. While training is a key aspect, we aimed to capture other ways technology influences SA and DMS (eg, real-time data feeds, decision support systems used in the field, and psychological impacts of constant connectivity).

### Databases

To comprehensively explore the scientific literature, this review used PubMed, Scopus, and WoS databases, each offering distinct advantages. PubMed, crucial for biomedical research, provides access to clinical and health-related studies. Scopus, with its interdisciplinary coverage, facilitated the identification of research trends and influential studies across diverse fields. Finally, WoS, known for its rigorous indexing, ensured the inclusion of high-impact research, including ethical and social perspectives.

### Search String

Two similar search strings (mentioned below) were used for each database and Google Scholar. SA and DMS were isolated to curtail the results and obtain eligible studies. Using SA and DMS together decreased the number of hits.

Disaster AND “Technology” AND “Situational Awareness”

Disaster AND “Technology” AND “Disaster Mindset”

### Inclusion Criteria

Studies in English discussing the impact of SA and DSM in disasters and public health emergencies between 2005‐ and 2025 were included.

### Exclusion Criteria

Studies not discussing the use of technology or unrelated to the educational aspects of disaster medicine, and those that did not follow the inclusion criteria or were in other languages, were excluded.

### Eligibility of Included Studies

Scientific or nonscientific studies that discussed the keywords in English and followed the inclusion criteria were all eligible for inclusion.

### Data Extraction

Data were extracted based on a predesign template by 2 of the authors and were validated by a third one. Data from included sources were charted using calibrated forms developed and piloted by the review team prior to use and consisted of title, date of publication, journal, study setting, and key points. This ensured consistency and accuracy in data collection. Data charting was performed independently during the review process, described above. Where necessary, efforts were made to contact original investigators to clarify or confirm data points, adhering to established systematic review guidelines.

### Review Process

The review process consisted of 2 stages: initial screening of titles and abstracts, followed by a second review and detailed examination of selected full-text studies, from which data were extracted for inclusion in the results. Three reviewers (AK-M, JLS, and MR-J), one trauma and disaster medicine specialist, one crisis manager, and one technology specialist examined the compiled studies independently to achieve unanimous consensus on inclusion. Any disagreement was resolved by consulting a fourth reviewer.

### Content Analysis

The outcomes were divided into diverse themes using conceptual content analysis, based on the similarities and differences [35]. The process includes defining the research question, coding the text, deciding the level of analysis, and developing rules for consistency. Finally, conclusions were drawn based on the patterns and trends identified.

## Results

### Overview

The search in scientific databases resulted in 147 studies from the following databases: PubMed (n=33), Scopus (n=21), and WoS (n=93). The Google Scholar search resulted in numerous documents. After sorting by relevance, the first 70 hits were added to the review list. Of the total number of 217 documents, in the first step, duplicates (n=5) and irrelevant papers (n=143) were removed by reviewing the titles and abstracts of compiled studies. Sixty-nine studies were considered relevant and included in the second review round by 3 reviewers. Each reviewer studied the included papers separately and selected the final number of studies to be included in this review (MultimediaAppendices 1  2 show the strategy and findings of each database and Google Scholar).

**Figure 1.**
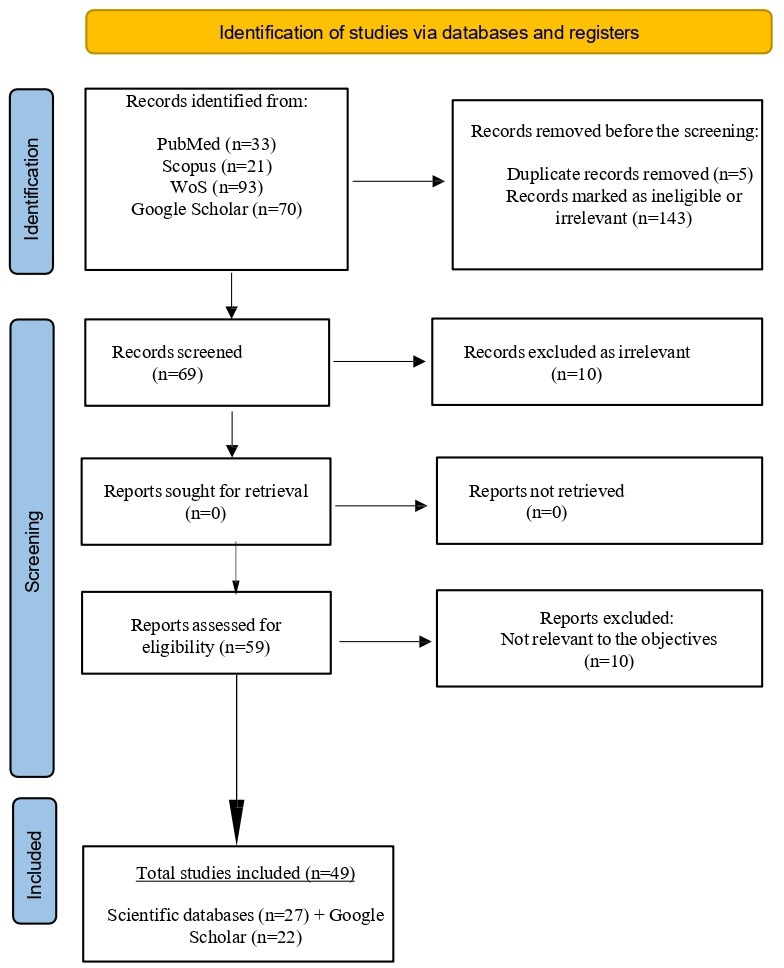
The search and literature selection process according to the PRISMA (Preferred Reporting Items for Systematic reviews and Meta-Analyses) flowchart for scoping reviews.

Ten irrelevant studies were removed before eligibility assessment. An additional 10 studies were not eligible, and the remaining 49 studies [24,25,36-82] were unanimously selected and included in this study at the final stage (Table 1; Figure 1). The search result consisted of peer-reviewed articles, policy documents, blogs, and guidelines.

**Table 1.** The setting and key points of the included studies, presented after the in-text citation.

Study details and reference number	Study setting	Key points
Mohsin et al (2016) [24]	General disaster response	Focuses on innovative systems for SA^a^
Su et al (2022) [25]	Global COVID-19 pandemic	Emphasizes the importance of a proactive DMS^b^
Naor and Laor (2020) [36]	Puerto Rico, Hurricane Maria recovery	Endsley’s model assesses SA in disaster recovery
Kettelhut et al (2017) [37]	Tactical biosurveillance	Visualizations impact biosurveillance SA
Glick and Barbara (2013) [38]	General disaster response	Examines the transition from awareness to decision-making
Jokela et al (2012) [39]	Major incidents	Evaluate RFID^c^ technology for improved situational awareness
McCurdy et al (2005) [40]	Medical disaster response	Explores video streaming for enhanced medical response
Kedia et al (2022) [41]	General disaster response	Reviews technologies for SA
Saadou and Chenji (2018) [42]	Disaster response networks	Presents research methods to optimize SA
Yuan et al (2013) [43	Disaster reliefs	Surveys how human sensor networks improve SA
Karavarsamis et al (2022) [44]	Disaster sites, adverse weather	Deep learning improves image clarity for better SA
Jain et al (2021) [45]	Mass-gathering events	Compares UAV^d^ effectiveness to standard practices in scene assessment
Jain et al (2018) [46]	Mass casualty incidents	Compares UAV effectiveness to standard practices in hazard identification
Lee et al (2022) [47]	Unmanned aerial vehicles	Explores deep learning for UAV-based human posture recognition
Mogaka et al (2024) [48]	Aerial scene image classification	Created a lightweight deep learning model for aerial image classification
Spandonidis et al (2021) [49]	Air cargo operations	Describes a data-driven system for situational awareness
Yuan et al (2022) [50]	China	Describes the key technologies used in China’s emergency response platforms
Tapia et al (2016) [51]	Public universities	Improves 911 texting for large-scale disasters
Gargees et al (2016) [52]	Incident support	Describes the use of visual cloud computing and SDN^e^ to support incident response
Chan and Purohit (2020) [53]	Public health systems during disasters	Highlights the challenges of using social media data for public health
Abid et al (2021) [54]	General disaster management	Explores AI’s^f^ role in improving disaster management
Panah et al (2024) [55]	General disaster zones	Social network analysis enhances disaster health care and resilience
Pekar et al (2020) [56]	General disaster zones, social media	Studies the use of social media for early disaster detection
Zafar et al (2024) [57]	Search and Rescue operations	Uses gesture control and deep learning to enhance unmanned ground vehicle performance in SAR^g^
Lenert et al (2011) [58]	Mass casualty settings	Focuses on wireless EHR^h^ systems in field care
Gao et al (2014) [59]	General emergency settings	Focuses on measuring and analyzing information flow
Gandhi et al (2021) [60]	Educational setting	Pandemic simulations improve student preparedness
Stroud et al (2010) [61]	Workshop and conceptual setting	Emphasizes the importance of SA in managing medical surge capacity during disasters. Discusses frameworks and challenges in achieving SA
Iserson KV [62]	Conceptual setting	Discusses the integration of various disaster response tools to empower clinical leadership and improve decision-making and coordination
O'Brien et al (2020) [63]	Conceptual setting. Multiagency emergency response	Explores the complexities and identifies challenges of achieving shared SA across different agencies during emergency response
Schwarz et al (2023) [64]	Case study	Investigates the potential for automating SA reporting in disaster management
Zhai (2022) [65]	Case study	Identifies disaster-related tweets to propose a structured, multi-layered approach for extracting actionable and relevant SA specifically tailored to the diverse informational needs of disaster responders and humanitarian organizations
Lowe et al (2016) [66]	Emergency medicine training	Explores the concept of SA within emergency medicine, focusing on developing a shared mental model among team members to enhance training and assessment methods
Khairilmizal et al (2023) [67]	Conceptual	Emphasizes effective disaster management, particularly in a sustainable context, hinges on robust strategies in 3 interconnected areas: Communication, Awareness, and Resource Management
De Monnin (2024) [68]	Conceptual and educational	Directly addresses the question of whether SA is an innate ability or a teachable skill. Likely explores pedagogical approaches and strategies for training SA
Westman et al (2024) [69]	Review of existing literature	Scopes the literature, and identifies SA as highly likely to be a prominent nontechnical skill
American College of Emergency Physicians (2014) [70]	Educational and informative	Outlines key competencies and identifies that SA is a fundamental competency in disaster response and likely included
Sapateiro and Antunes (2009) [71]	Conceptual	Proposes an emergency response model aimed at improving and enhancing SA
Lateef F (2022) [72]	Emergency medicine training	Explores the interconnectedness of SA, clinical reasoning, and clinical judgment in emergency medicine, advocating for cross-training using case-based discussions
Levin and Sauer (2012) [73]	Emergency medicine	Discusses the concept and importance of SA specifically within the context of emergency medicine. Likely covers its application and challenges in this setting
Bouzidi et al (2022) [74]	Case study (COVID-19)	Uses the COVID-19 pandemic as a case study to demonstrate how warning systems, SA, assessment methods, and education can be enhanced for emergency management
Lambert et al (2021) [75]	Technological development (AI)	Focuses on the application of AI to enhance SA during disaster response, discussing potential benefits and challenges
The Hospital Health System Association of Pennsylvania (2025) [76]	Policy and practical guidelines	Discusses the continued relevance of SA for hospitals and health care systems
Government Technology Handbook (2022) [77]	Policy and conceptual setting	Advocates for a new mindset in emergency response, integrating technology, data, and collaborative approaches to improve overall effectiveness, including SA
Kuziemsky et al (2012) [78]	Information system design	Proposes an “upstream-downstream” approach for designing disaster management information systems, likely emphasizing the flow of information to support SA and decision-making
Laurila-Pant et al (2012) [79]	Simulation research methodology	Proposes a structured method for evaluating how well different agencies or teams develop a common understanding of a disaster situation and how this impacts their joint decision-making during simulated disaster exercises
Cooper et al (2013) [80]	Review of existing literature	Systematically reviews the various tools and methods used to measure SA in emergency settings, as well as the outcomes associated with SA
Williams et al. (2022) [81]	Paramedicine education	A pilot study investigating the impact of immersive simulation on paramedicine students’ SA, using a mixed methods approach to assess its effectiveness
Gu et al (2018) [82]	Model development (Sensors)	Proposes a model for health and safety, SA, and emergency management that leverages multisensor signal fusion to gather comprehensive data and enhance understanding

a SA: situational awareness.

b DMS: disaster mindset.

c RFID: Radio Frequency Identification.

d UAV: unmanned aerial vehicle.

e SDN: software-defined networking.

f AI: artificial intelligence.

g SAR: Search and Rescue.

h EHR: electronic health record.

### The Summary of the Results

Across the compiled documents, a consistent emphasis emerged on the pivotal role of SA in enhancing emergency response across various domains, including medical emergencies, disaster management, and multiagency operations. The number of documents discussing DMS was limited. These studies [24,25,36-82] indicate that access to real-time, accurate, and integrated data is fundamental (Table 1). Technologies such as unmanned aerial vehicles (UAVs), AI, and the Internet of Things (IoT) are transforming disaster response. However, the success of any system hinges on its usability and relevance to end users. Optimizing information flow, leveraging AI’s growing role, and prioritizing real-time processing are vital for enhancing SA and improving outcomes in critical situations [24,25,36-82].

While technology is crucial in providing access to real-time information, it must be combined with purposeful training, clear communication, and an understanding of human cognitive factors. Furthermore, the transition from simply having information to making effective decisions is a critical area of focus. By addressing the challenges and leveraging the opportunities presented by technology and research, the ability to respond can significantly improve and mitigate the impact of disasters. Furthermore, the studies underscore the importance of integrating SA with other crucial skills such as clinical reasoning, communication, and teamwork, particularly in high-stress environments. Developing a DMS and shared mental models among responders is essential for effective coordination and adaptability. The research also reveals the limitations of traditional SA measurement methods and advocates for adopting comprehensive, mixed methods approaches to accurately assess and improve SA. Ultimately, the cumulative data advocate for a holistic, technology-enhanced, and competency-based strategy to cultivate and maintain SA, leading to improved emergency response outcomes and enhanced safety for both responders and affected populations [24,25,36-41,41-82].

This necessitates developing and implementing robust SA frameworks, tools, and training programs to ensure prompt and accurate information dissemination, thereby facilitating informed decision-making and efficient resource allocation. Technological advancements, such as AI-driven systems, multisensor signal fusion, and immersive simulations, are identified as critical components in improving SA by providing real-time data and enhancing responder capabilities. Only a few studies in the included publications discussed a DMS [25,77], explicitly addressing the concept of a “disaster readiness mindset,” which inherently involves proactivity. The COVID-19 pandemic was used as a case study to emphasize the importance of being prepared and proactive in the face of potential disasters.

## Discussion

### Principal Findings

While the scientific studies consistently demonstrate that enhancing SA is paramount for effective disaster response and emergency management, the online reviews of several ongoing programs do not provide enough evidence on the extent to which these programs include the concepts of SA and a DMS. Most programs, however, touch both concepts indirectly by considering psychological, communicational, and decision-making aspects of disaster management. Table 2 provides information about a few current programs that seem to include SA and a DMS in their curricula [26-32].

**Table 2.** A short description of some disaster medicine courses that likely include situational awareness and a disaster mindset in their programs.

No. (Country)	Title	Description
1 (Spain, Sweden, Cyprus)	Erasmus Mundus Master Program - Public Health in Disasters (University of Oviedo, Karolinska Institutet, University of Nicosia).	A 2-year master’s program, including “Global Health and Disasters” and “Public Health Response in Health Crisis and Disasters,” which likely touches upon the psychological and contextual aspects of disaster response [26].
2 (Italy)	Training Disaster Medicine Trainers (TdmT) - IFMSA and CRIMEDIM (University of Piemonte Orientale).	Offers medical students the knowledge and skills in disaster medicine and global health, including topics such as complex humanitarian emergencies and disaster risk management. While the description doesn’t explicitly mention “disaster mindset,” the focus on humanitarian emergencies suggests the inclusion of psychological preparedness and situational awareness [27].
3 (Denmark)	Master of Disaster Management - University of Copenhagen.	Includes courses such as “Preparedness and Response to Humanitarian Crises” and “Disaster Risk Management: From Theory to Practice,” likely involving elements of understanding the disaster environment and its psychological challenges [28].
4 (U.S.A.)	Disaster Fellowship - Johns Hopkins Department of Emergency Medicine.	This fellowship includes public health training and disaster field work, implying the development of skills to understand and navigate complex disaster scenarios, which include situational awareness and mindset [29].
5 (U.S.A.)	Center for Disaster Medicine - New York Medical College.	This center offers operational and tactical medicine training, including elements of decision-making under stress and maintaining situational awareness in chaotic environments [30].
6 (Sweden)	PRAD-MED – Preparedness and Disaster Medicine (Sahlgrenska University Hospital and Gothenburg University)	This 6-week-long course, spread over a year, includes global, national, and regional aspects of disaster and public health emergencies’ preparedness and management. It has a mix of international and national experts and particularly discusses the transition of care from a multicasualty perspective to mass casualty and disaster casualty scenarios. It is a hybrid course using tabletop exercises and modular simulation training [31].
7 (Italy, Belgium)	The European Master in Disaster Medicine (EMDM)	This is a 1-year, blended Advanced Master of Science program for health professionals. It combines a self-directed online course with a 2-week intensive residential session featuring debates and complex simulations (including VR)^a^, culminating in a final online exam and a research thesis. The EMDM^b^ aims to equip participants with comprehensive knowledge, practical skills, and a systemic approach to health disaster management, fostering competent and adaptable professionals for emergency and humanitarian health response through its competency-based, multidisciplinary, and internationally focused curriculum [32].

a VR: virtual reality.

b EMDM: European Master in Disaster Medicine.

This study indicates that effective disaster response hinges on real-time, accurate, and integrated data. While technology revolutionizes information flow, boosting SA and decision-making, it needs purposeful training, clear communication, and an understanding of human cognitive factors to translate data into action. Cultivating a DMS and shared mental models is crucial, as well as integration of SA with skills such as clinical reasoning and teamwork. Many programs indirectly touch on these concepts, but more explicit inclusion and evidence of integration are needed. Ultimately, a holistic, technology-driven, and competency-based approach is vital for enhanced emergency response and safety.

### The Evolving Landscape of SA in Disaster Management

SA, the ability to perceive, comprehend, and project the current state of a dynamic environment, has emerged as a critical factor across diverse domains because it enables individuals and teams to make informed decisions, anticipate potential problems, and respond effectively to dynamic situations, particularly in disaster response, medical emergencies, and biosurveillance. The papers reviewed in this study consistently underscore that mere data availability is insufficient; it must be transformed into actionable information, supporting informed decision-making and effective action [36-38,41,42].

Technology plays a pivotal role in enhancing SA. UAVs provide rapid visual information, offering comprehensive overviews and improved hazard identification. Radio Frequency Identification (RFID) enables real-time tracking of personnel and assets, enhancing accountability and safety. Effective information visualization tools are crucial for presenting complex data in an understandable format, especially during time-sensitive situations. Satellite imagery offers real-time wide-area information, while ubiquitous video, through technologies such as “RealityFlythrough,” allows responders to virtually navigate and assess disaster zones [39,40,44-52].

However, achieving optimal SA is not without its challenges. Information overload, particularly from social media, can overwhelm decision-makers. Ensuring data veracity, especially from diverse sources, remains a significant hurdle. The transition from awareness to action requires decision-makers to effectively translate information into timely and appropriate responses. Individual cognitive factors, such as stress and fatigue, can also impact SA [53].

Endsley 3-level model [36] (perception, comprehension, and projection) serves as a valuable framework for understanding and improving SA. Studies consistently use this model to analyze how individuals process information in complex environments. Furthermore, simulation exercises and training programs are essential for developing the necessary skills and experience to maintain SA, particularly when implementing new technologies [83].

Recent advancements in data-driven approaches and AI have further revolutionized SA. End-user centricity is paramount, ensuring systems are tailored to the specific needs and preferences of those using them. Data integration from diverse sources creates comprehensive situational understanding, especially in complex environments such as air cargo operations. AI’s transformative potential lies in its ability to process vast datasets, identify patterns, and provide predictive insights, enhancing early warning systems and resource allocation [44,47-50,54,55].

Human Sensor Networks highlight the value of citizen participation in gathering real-time, localized data, complementing traditional sensor networks. Technology integration, encompassing UAVs, GIS, remote sensing, social media, IoT, and AI, is essential for a unified situational view. Optimizing information flow within response networks, enhancing end-user centricity, and leveraging UAVs for scene assessment are critical for effective disaster management. Scaling 911 texting, using social media for early detection, and integrating deep learning with unmanned ground vehicles further enhance response capabilities. China’s emergency platforms exemplify the integration of advanced technologies for comprehensive SA [51,52,56-58].

Deep learning for UAVs, visual cloud computing with software-defined networking, and ultralightweight deep learning models such as TinyEmergencyNet improve real-time visual information processing. Chebyshev Transform-based trajectory prediction enhances accuracy in apps such as autonomous vehicles [48,49].

### Cultivating a Resilient DMS: Synthesis of Key Attributes

The concept of a “disaster mindset” emerges as a crucial element in navigating the complexities of modern crises [25]. It transcends mere reaction, advocating for a proactive, adaptable, and informed approach to preparedness and resilience. Analyzing the relevant literature reveals a set of key attributes that define this critical mindset.

A fundamental aspect of a DMS is the ability to anticipate potential threats and vulnerabilities. A DMS shifts the focus from reactive responses to proactive measures and involves moving beyond reactive measures and implementing preventative strategies. Performance-based infrastructure resilience studies exemplify this, showcasing the importance of evaluating resilience-enhancing options before a disaster strikes [84].

Disasters often present diverse challenges, requiring flexible and responsive strategies. Cross-cultural mood perception research underscores the need to recognize and adapt to varying cultural responses during crises, demonstrating that empathy and understanding are integral to effective support [1,85]. A DMS is characterized by a commitment to continuous learning from past events, both successes and failures. The analysis of the Great East-Japan Earthquake emphasizes the importance of incorporating historical lessons into current preparedness strategies, ensuring that past mistakes are not repeated [86].

In an increasingly interconnected and digital world, vigilance against evolving threats is essential. The study on cybersecurity in the SADC region highlights the need to adapt to new technologies and address emerging vulnerabilities, particularly in the realm of cyberattacks. A core component of a DMS is the commitment to building robust systems and communities that can withstand shocks and recover quickly. This includes the ability to construct resilient infrastructure and foster community resilience. A DMS is grounded in data and facts. It is crucial to have the ability to gather, analyze, and use information to make the best possible decisions. This ensures that responses are based on accurate and timely information [87-89].

### Integration of Technology and Mindset

The effective integration of technology into disaster management is contingent upon a robust DMS. While technology provides powerful tools for early warning, SA, and response, it is the proactive, adaptable, and informed mindset that ensures these tools are used effectively. For example, AI can enhance predictive capabilities, but a DMS ensures that these predictions are used to inform proactive measures and build resilience. The DMS is not static; it must evolve in response to changing threats and technological advancements. The COVID-19 pandemic significantly increased cybersecurity awareness, highlighting the necessity for a flexible and responsive approach [25,88]. Similarly, the increase in climate-related disasters underscores the necessity for an evolving mindset that can address new and emerging challenges. Cultivating a resilient DMS is crucial for navigating the complexities of modern crises. It involves a proactive, adaptable, and informed approach to preparedness and resilience [89,90]. By embracing these key attributes, individuals, organizations, and communities can better anticipate, respond to, and recover from disasters, ultimately building a safer and more resilient future.

### Using Technology to Fill in the Gaps in Disaster Simulation Exercises

Disaster exercises primarily focus on the core pillars of disaster medicine, including planning, response, safety, triage, clinical competence, psychological first aid, interdisciplinary collaboration, and continuous improvement. Nevertheless, the main challenge is to capture the dynamic of an incident that may transition from a multiple casualty to a mass casualty and further to a disaster casualty event. As emergencies escalate, this transition unfolds like increasingly challenging chapters in crisis response [19,90,91].

In a multiple casualty incident, numerous injured individuals are involved. Local emergency services, including paramedics, firefighters, and police, swiftly arrive to triage the injured and prioritize those needing immediate medical attention. The local hospital prepares to receive patients, and the situation remains manageable within the city’s emergency resources [92,93]. In a mass casualty incident, the number of casualties quickly overwhelms local emergency services. Regional and national emergency response plans are activated, bringing additional medical teams, equipment, and supplies from neighboring areas. Coordination becomes complex as multiple agencies work together, focusing on systematic triage to use resources efficiently and save lives [94]. Finally, in a disaster casualty situation, a catastrophic event devastates an entire region, affecting infrastructure, homes, and critical services. The number of casualties is overwhelming, exhausting local and regional resources. A comprehensive, multisectoral approach is required, involving national disaster response plans and international aid. Coordination expands to include government agencies, nongovernmental organizations (NGOs), international relief organizations, and community leaders, addressing immediate medical care and long-term recovery and rebuilding efforts [94,95].

Throughout these transitions, the DMS and SA evolve. In a multiple casualty incident, the focus is on rapid response and localized resource management. As the situation escalates to a mass casualty incident, the response becomes scalable, requiring broader coordination and systematic triage. Finally, in a disaster casualty situation, the response is comprehensive, involving strategic coordination across multiple sectors and long-term planning. While current simulation exercises in disaster medicine are invaluable for training and preparedness, they have several gaps, which influence the outcomes of simulation exercises and the development of SA and DMS in individuals and collectively [38,91-95].

These gaps must be addressed to enhance their effectiveness. Table 3 shows the gaps in disaster medicine simulation and the ideal technological solutions. These solutions can help close the gaps in current disaster medicine simulation exercises, enhancing the training and preparedness of health care professionals [95-98].

**Table 3.** Outlines the gaps in disaster medicine simulation and the corresponding technological solutions.

Gaps	Description	Technological solutions
Lack of uniform standards	There is a need for standardized procedures and protocols across different simulation exercises to ensure consistent training outcomes.	Advanced simulation software to standardize procedures and protocols
Insufficient rigorous evaluation	Many exercises lack rigorous evaluation methods to comprehensively assess their effectiveness, making it difficult to identify areas for improvement.	AI^a^ for automated evaluation and detailed feedback
Limited feedback mechanisms	Immediate and constructive feedback is crucial for learning, but some exercises do not provide adequate feedback to participants.	AI-driven systems for real-time, constructive feedback
Limited realism	Some simulations may not fully capture the complexity and unpredictability of actual disasters, limiting participants’ preparedness for real-life situations.	VR^b^ and AR^c^ for highly realistic and immersive disaster scenarios
Scenario diversity	There is often a lack of diverse scenarios covering a wide range of potential disasters, including rare but high-impact events.	VR and AR to simulate a wide range of disaster scenarios
Gaps in interagency coordination	Effective disaster response requires seamless coordination between various agencies and disciplines, which some exercises do not adequately address.	Telemedicine platforms for seamless communication and coordination
Limited resources	High-quality simulation exercises can be resource-intensive, requiring significant time, personnel, and financial investment, limiting the frequency and scope of training.	mHealth apps for efficient resource management and cost-effective training
Underuse of advanced technologies	Technologies such as VR and AR have the potential to enhance simulation training but are not yet widely integrated into disaster medicine education.	Blended learning platforms, combining online modules with hands-on simulations

a AI: artificial intelligence.

b VR: virtual reality.

c AR: augmented reality.

### Fostering SA and Developing a DMS

While UAVs, GIS, AI, IoT, and social media monitoring offer significant capabilities, the optimal technology for enhancing SA in disaster management is context dependent. A well-integrated system combining these tools, with a strong emphasis on data management and user-centered design, is likely to be most effective. Furthermore, technologies such as VR and AR simulations and online educational platforms can cultivate a proactive DMS through realistic training and widespread dissemination of SA. Data visualization promotes data-driven preparedness, while reliable communication and alert systems ensure timely information sharing. Social media and crowdsourcing can also foster community resilience and shared responsibility. However, the successful adoption and impact of these technologies, crucial for building a robust DMS, hinge on accessibility, information accuracy, and adequate user training [25,45,46,51,53,96-101].

Indeed, research in information systems underscores the critical role of user adoption, often explained by the Technology Acceptance Model (TAM), which posits that perceived usefulness and ease of use are key determinants of whether technologies are embraced or discarded [102].

Finally, effective education for SA and DMS must adapt to diverse cultural, social, and economic contexts. This means respecting local beliefs, integrating traditional knowledge, and using community leaders as trusted communicators, often through visual or oral methods. Socially, programs should be co-created with communities, leverage existing networks, and explicitly address vulnerabilities of marginalized groups to build trust and inclusivity [103]. Furthermore, the type of disaster significantly impacts educational needs. For slow-onset events such as drought or sea-level rise, initiatives focus on long-term preparedness, sustainable practices, and climate adaptation. For sudden-onset events such as earthquakes or tsunamis, the emphasis shifts to immediate evacuation protocols, early warning system comprehension, and rapid decision-making skills. Education for man-made disasters (eg, industrial accidents and terrorism) would include understanding specific hazards, lockdown procedures, or emergency contact protocols, emphasizing different aspects of SA and mindset than natural hazards [104,105]. Table 3 shows the gaps in disaster education and how technology can be used as a solution.

### Limitations

Unlike systematic reviews, this study did not assess the quality or risk of bias of the included studies. This means that some findings, while providing an overview, might be influenced by methodologically weaker research.

As a scoping review, the aim of this study was to map the breadth of available literature rather than delve into the in-depth nuances of individual studies or provide definitive answers on intervention effectiveness. While we used a systematic process, and 3 researchers mitigated potential subjectivity during study selection and data extraction, the broad inclusion of diverse methodologies, populations, and outcomes naturally presents challenges in synthesizing findings into a singular quantitative or qualitative conclusion.

A significant limitation arises from the sole inclusion of studies in English. Due to this language restriction, valuable insights from non-English literature might have been missed. With the increasing sophistication and accessibility of AI translation tools, relying solely on English-language publications could be a substantial limitation. While AI translation is improving, it still grapples with accurately capturing idiomatic expressions, cultural nuances, and complex contextual meanings, particularly in specialized fields such as disaster medicine. Therefore, the absence of non-English studies, even with the potential of AI translation, could introduce a language and cultural bias, potentially limiting the comprehensiveness and global generalizability of this review’s findings.

### Future Directions

The future of leveraging technology for SA and DMS is heading toward deep integration, automation, and intelligent systems, shifting us to a more proactive and adaptive approach in disaster management [105]. Key trends include hyperenhanced SA through ubiquitous IoT sensors and AI-powered data analysis, allowing for real-time prediction and actionable insights. Immersive visualization via AR, VR, and MR will revolutionize how decision-makers perceive disaster environments, while digital twins will enable dynamic scenariosimulations and optimized resource allocation [13,96].

Crucially, technology will also foster a more proactive and adaptive DMS. This involves personalized preparedness through wearables and AI, alongside immersive training in VR and AR simulations that build psychological resilience. AI-augmented decision support will act as an intelligent copilot for emergency managers, ensuring seamless human-AI collaboration. While challenges such as interoperability, data security, and ethical considerations remain, the overarching goal is to create a highly interconnected, intelligent, and immersive technological ecosystem that significantly enhances our collective ability to anticipate, respond to, and recover from disasters [13,96].

### Conclusions

In conclusion, the role of technology in enhancing situational awareness and fostering a robust DMS is unequivocal yet necessitates careful consideration. While tools such as UAVs, AI, and the IoT offer unprecedented data and analytical capabilities, their effectiveness is contingent upon user-centered design, data integrity, and seamless integration. Importantly, cultivating a proactive DMS extends beyond the mere deployment of technology. VR and AR simulations, educational platforms, and communication systems are instrumental in developing preparedness, resilience, and informed decision-making. Ultimately, the successful application of technology in disaster management requires a holistic approach that integrates advanced tools with human-centered design, effective information management, and a commitment to continuous learning. Moving beyond simply equipping individuals and teams with technical solutions, the true significance lies in their ability to use these tools effectively, ensuring they are also mentally prepared to navigate the complexities of crises and can seamlessly integrate technology into their response strategies.

## Supplementary material

10.2196/75404 Multimedia Appendix 1 Search results from scientific databases.

10.2196/75404 Multimedia Appendix 2 Search results from Google Scholar search.

10.2196/75404 Checklist 1 PRISMA-ScR checklist.

## Abbreviations

AI: artificial intelligence
AR: augmented reality
DMS: disaster mindset
GIS: geographic information systems
IoT: Internet of Things
NGO: nongovernmental organization
RFID: Radio Frequency Identification
SA: situational awareness
TAM: Technology Acceptance Model
UAV: unmanned aerial vehicles
VR: virtual reality
