# Supplementary material for: Cultivating Disaster Preparedness: Scoping Review of Technology’s Contribution to Situational Awareness and Disaster Mindset in Disaster Medicine
Source: Online J Public Health Inform. 2025 Oct 10;17:e75404. doi: 10.2196/75404 (PMC12513712; doi:10.2196/75404)
Supplement: Multimedia Appendix 1 [file ojphi-v17-e75404-s001.pdf]

Appendix 1: Search results from scientific databases

All included papers from Scientific databases (n=27)

1. A data-driven situational awareness system for enhanced air cargo operations emergency control
2. A Survey of Deep Learning-Based Image Restoration Methods for Enhancing Situational Awareness at Disaster Sites: The Cases of Rain, Snow and Haze.
3. An Innovative System to Enhance Situational Awareness in Disaster Response: What are End Users Looking for in Such Systems
4. Challenges to Transforming Unconventional Social Media Data into Actionable Knowledge for Public Health Systems During Disasters.
5. Comparison of Unmanned Aerial Vehicle **Technology** versus Standard Practice of Scene Assessment by Paramedic Students of a Mass-Gathering Event.
6. Comparison of Unmanned Aerial Vehicle **Technology** Versus Standard Practice in Identification of Hazards at a Mass Casualty Incident Scenario by Primary Care Paramedic Students.
7. Deep Learning-Based Human Body Posture Recognition and Tracking for Unmanned Aerial Vehicles
8. Design and evaluation of a wireless electronic health records system for field care in mass casualty settings.
9. Disaster Health Care and Resiliency: A Systematic Review of the Application of Social Network Data Analytics.
10. Disaster recovery after Hurricane Maria in Puerto Rico: Assessment using Endsley's three-level model of situational awareness.
11. Early Detection of Heterogeneous Disaster Events Using Social Media
12. Empirical Study on the Impact of a Tactical Biosurveillance Information Visualization on Users' Situational Awareness.
13. Enhancing unmanned ground vehicle performance in SAR operations: integrated gesture-control and deep learning framework for optimised victim detection
14. Harness Human Sensor Networks for Situational Awareness in Disaster Reliefs: A Survey
15. Implementation and evaluation of a pandemic simulation exercise among undergraduate public health and nursing students: A mixed-methods study.
16. Incident-Supporting Visual Cloud Computing Utilizing Software-Defined Networking
17. Increased situation awareness in major incidents-radio frequency identification (RFID) technique: a promising tool.
18. Key technologies of the emergency platform in China
19. Moving from situational awareness to decisions during disaster response: transition to decision making.
20. Optimizing Situational Awareness in Disaster Response Networks
21. Quantifying information flow during emergencies.
22. RealityFlythrough: enhancing **situational awareness** for medical response to **disasters** using ubiquitous video.
23. Scaling 911 Texting for Large-Scale Disasters: Developing Practical Technical Innovations for Emergency Management at Public Universities

24. Technologies Enabling Situational Awareness During Disaster Response: A Systematic Review
25. The need for a **disaster** readiness **mindset**: A key lesson from the coronavirus disease 2019 (COVID-19) pandemic.
26. TinyEmergencyNet: a hardware-friendly, ultra-lightweight deep learning model for aerial scene image classification
27. Toward an integrated disaster management approach: How artificial intelligence can boost disaster management

Scientific databases

PubMed

SA

Disaster AND "Technology" AND "Situational awareness" = 29 hits, 18 included

Link to all studies

https://pubmed.ncbi.nlm.nih.gov/?term=Disaster%20AND%20%22Technology%22%20AND%20%22Situational%20awareness%22&sort=date&page=3

1. **Disaster** Health Care and Resiliency: A Systematic Review of the Application of Social Network Data Analytics. Rasouli Panah H, Madanian S, Yu J.Disaster Med Public Health Prep. 2025 Jan 3;18:e334. doi: 10.1017/dmp.2024.294.
2. Enhancing unmanned ground vehicle performance in SAR operations: integrated gesture-control and deep learning framework for optimised victim detection. Zafar MH, Moosavi SKR, Sanfilippo F.Front Robot AI. 2024 Jun 18;11:1356345. doi:10.3389/frobt.2024.1356345. eCollection 2024. Seems to be on technological development of censors in UGVs and not about the situational awareness of EM staff members.
3. A Survey of Deep Learning-Based Image Restoration Methods for Enhancing **Situational Awareness** at **Disaster** Sites: The Cases of Rain, Snow and Haze. Karavarsamis S, Gkika I, Gkitsas V, Konstantoudakis K, Zarpalas D.Sensors (Basel). 2022 Jun 22;22(13):4707. doi: 10.3390/s22134707.
4. Comparison of Unmanned Aerial Vehicle **Technology** versus Standard Practice of Scene Assessment by Paramedic Students of a Mass-Gathering Event. Jain T, Sibley A, Stryhn H, Lund A, Hubloue I.Prehosp Disaster Med. 2021 Dec;36(6):756-761. doi: 10.1017/S1049023X2100114X. Epub 2021 Oct 27.PMID: 34702422 Clinical Trial.
5. Digital Strategies to Improve Food Assistance in **Disasters**: A Scoping Review. Martin NM, Sundermeir SM, Barnett DJ, van Dongen EJI, Rosman L, Rosenblum AJ, Gittelsohn J.Disaster Med Public Health Prep. 2021 Oct 11;17:e40. doi: 10.1017/dmp.2021.281.
6. Implementation and evaluation of a pandemic simulation exercise among undergraduate public health and nursing students: A mixed-methods study. Gandhi S, Yeager J, Glaman R.Nurse Educ Today. 2021 Mar;98:104654. doi: 10.1016/j.nedt.2020.104654. Epub 2020 Nov 7. This is about the effect of simulation

exercises on situational awareness, rather than the effects of technology on situational awareness.

7. **Disaster** recovery after Hurricane Maria in Puerto Rico: Assessment using Endsley's three-level model of **situational awareness**. Naor M, Laor E.J Bus Contin Emer Plan. 2020 Jan 1;13(3):278-288.
8. Challenges to Transforming Unconventional Social Media Data into Actionable Knowledge for Public Health Systems During **Disasters**. Chan JL, Purohit H.Disaster Med Public Health Prep. 2020 Jun;14(3):352-359. doi: 10.1017/dmp.2019.92. Epub 2019 Oct 15.
9. Comparison of Unmanned Aerial Vehicle **Technology** Versus Standard Practice in Identification of Hazards at a Mass Casualty Incident Scenario by Primary Care Paramedic Students. Jain T, Sibley A, Stryhn H, Hubloue I.Disaster Med Public Health Prep. 2018 Oct;12(5):631-634. doi: 10.1017/dmp.2017.129. Epub 2018 Jan 31.
10. A heuristic approach to global landslide susceptibility mapping. Stanley T, Kirschbaum DB.Nat Hazards (Dordr). 2017 May;87(1):145-164. doi: 10.1007/s11069-017-2757-y. Epub 2017 Feb 7.
11. Recent field experiments with commercial satellite imagery direct downlink. Gonzalez AR, Amber SH.J Emerg Manag. 2017 Jan/Feb;15(1):62-66. doi: 10.5055/jem.2017.0313. Seems to be more about using technology to access data and information than enhancing the capacity of EM staff to create situational awareness.
12. Empirical Study on the Impact of a Tactical Biosurveillance Information Visualization on Users' **Situational Awareness**. Kettelhut VV, Vanschooneveld TC, McClay JC, Mercer DF, Fruhling A, Meza JL.Mil Med. 2017 Mar;182(S1):322-329. doi: 10.7205/MILMED-D-16-00143.
13. Implementation and modeling of a Regional Hub Reception Center during mass evacuation operations. Wojtalewicz C, Kirby A, Dietz JE.J Emerg Manag. 2014 May-Jun;12(3):197-210. doi: 10.5055/jem.2014.0172.
14. Moving from **situational awareness** to decisions during **disaster** response: transition to decision making. Glick JA, Barbara JA.J Emerg Manag. 2013 Nov-Dec;11(6):423-32. doi: 10.5055/jem.2013.0155. Technology does not seem to be covered.
15. Quantifying information flow during emergencies. Gao L, Song C, Gao Z, Barabási AL, Bagrow JP, Wang D.Sci Rep. 2014 Feb 6;4:3997. doi: 10.1038/srep03997. Technology is not studied as a means to create situational awareness.
16. Increased situation awareness in major incidents-radio frequency identification (RFID) technique: a promising tool. Jokela J, Rådestad M, Gryth D, Nilsson H, Rüter A, Svensson L, Harkke V, Luoto M, Castrén M.Prehosp Disaster Med. 2012 Feb;27(1):81-7. doi: 10.1017/S1049023X12000295. Epub 2012 Apr 25. About technology as a facilitator of the availability of information and data.
17. Design and evaluation of a wireless electronic health records system for field care in mass casualty settings. Lenert LA, Kirsh D, Griswold WG, Buono C, Lyon J, Rao R, Chan TC.J Am Med Inform Assoc. 2011 Nov-Dec;18(6):842-52. doi: 10.1136/amiajnl-2011-000229. Epub 2011 Jun 27. About technology as a facilitator of the availability of information and data.
18. RealityFlythrough: enhancing **situational awareness** for medical response to **disasters** using ubiquitous video. McCurdy NJ, Griswold WG, Lenert LA.AMIA

Annu Symp Proc. 2005;2005:510-4. Seems to be more about developing technology to handle data than enhancing the capacity of EM staff to create situational awareness.

DSM

Disaster AND Technology AND disaster mindset = 6 hits, 1 included

Link to all papers

https://pubmed.ncbi.nlm.nih.gov/?term=Disaster+AND+%22Technology%22+AND+Disaster+mindset&sort=date

Selected studies

1. The need for a **disaster** readiness **mindset**: A key lesson from the coronavirus disease 2019 (COVID-19) pandemic. No abstract. Su Z, McDonnell D, Ahmad J.Infect Control Hosp Epidemiol. 2022 Apr;43(4):538-539. doi: 10.1017/ice.2021.26. Epub 2021 Jan 25.

Scopus

SA

Link to all studies

Disaster AND Technology AND Situational awareness: 20, FR 3 included

https://www-scopus-com.ezproxy.ub.gu.se/results/results.uri?st1=%22World+health+organization%22+AND+%22United+States%22+AND+%22Collaboration%22+AND+%22Global+Health%22&st2=&s=TITLE-ABS-KEY%28disaster+AND+%22technology%22+AND+%22situational%22+AND+%22awareness%22%29&limit=20&origin=resultslist&sort=plf-f&src=s&sot=b&sdt=cl&sessionSearchId=607ea8c929eab84c5a2869233217bcba&yearFrom=2015&yearTo=2024&cluster=scosubjabbr%2C%22SOCI%22%2Ct%2C%22DECI%22%2Ct%2Bscosubtype%2C%22re%22%2Ct%2C%22ar%22%2Ct needs GU login to access

Selected studies, first review

1. Toward an integrated disaster management approach: How artificial intelligence can boost disaster management
2. A data-driven situational awareness system for enhanced air cargo operations emergency control
3. intelligent ULD; Structural health monitoring; fire suppression; locking status; human machine interface
4. An Innovative System to Enhance Situational Awareness in Disaster Response: What are End Users Looking for in Such Systems

DMS

Disaster AND "Technology" AND Disaster mindset= 1, FR 1 included

Link to all studies

https://www-scopus-com.ezproxy.ub.gu.se/results/results.uri?st1=%22World+health+organization%22+AND+%22United+States%22+AND+%22Collaboration%22+AND+%22Global+Health%22&st2=&s=TITLE-ABS-KEY%28disaster+AND+%22technology%22+AND+disaster+mindset%29&limit=20&origin=searchbasic&sort=plf-5,
f&src=s&sot=b&sdt=b&sessionSearchId=607ea8c929eab84c5a2869233217bcba&yearFrom=2015&yearTo=2024&cluster=scosubjabbr%2C%22SOCI%22%2Ct%2C%22DECI%22%2Ct%2Bscosubtype%2C%22re%22%2Ct%2C%22ar%22%2Ct

1. The Potential of Digitally Enabled Disaster Education for Sustainable Development Goals

<u>Web of Science</u>

SA

Disaster AND Technology AND Situational awareness =80, FR 13 included

Link to all studies

https://www-webofscience-com.ezproxy.ub.gu.se/wos/woscc/summary/bab18f0c-a720-401a-a03c-1cb6bfe25341-0150b14d06/relevance/1

Selected studies in first review

1. Harness Human Sensor Networks for Situational Awareness in Disaster Reliefs: A Survey Disaster reliefHuman sensor networkSituational awarenessSocial media
2. Technologies Enabling Situational Awareness During Disaster Response: A Systematic Review
3. Optimizing Situational Awareness in Disaster Response Networks
4. An Innovative System to Enhance Situational Awareness in Disaster Response What are End Users Looking for in Such Systems
5. Comparison of Unmanned Aerial Vehicle Technology versus Standard Practice of Scene Assessment by Paramedic Students of a Mass-Gathering Event
6. Scaling 911 Texting for Large-Scale Disasters: Developing Practical Technical Innovations for Emergency Management at Public Universities
7. Early Detection of Heterogeneous Disaster Events Using Social Media
8. Enhancing unmanned ground vehicle performance in SAR operations: integrated gesture-control and deep learning framework for optimised victim detection
9. Key technologies of the emergency platform in China
10. Deep Learning-Based Human Body Posture Recognition and Tracking for Unmanned Aerial Vehicles
11. Incident-Supporting Visual Cloud Computing Utilizing Software-Defined Networking
12. TinyEmergencyNet: a hardware-friendly ultra-lightweight deep learning model for aerial scene image classification

13. Chebyshev Transform-Based Robust Trajectory Prediction Using Recurrent Neural Network

DMS

Disaster AND Technology AND Disaster mindset=18, FR 4

The link to 18 studies

https://www-webofscience-com.ezproxy.ub.gu.se/wos/woscc/summary/35480192-d9c5-4c7a-a351-52990635fb80-0150b1c076/relevance/1

First review selection

1. The impact of COVID-19 on cybersecurity awareness-raising and mindset in the southern African development community (SADC)
2. The Great East-Japan Earthquake and Devastating Tsunami: An Update and Lessons from the Past Great Earthquakes in Japan since 1923
3. Performance-based selection of pathways for enhancing built infrastructure resilience
4. Cross-Cultural Comparison of Mood Perception During Initial Pandemic Response
