# Supplementary material for: Cultivating Disaster Preparedness: Scoping Review of Technology’s Contribution to Situational Awareness and Disaster Mindset in Disaster Medicine
Source: Online J Public Health Inform. 2025 Oct 10;17:e75404. doi: 10.2196/75404 (PMC12513712; doi:10.2196/75404)
Supplement: Multimedia Appendix 2 [file ojphi-v17-e75404-s002.pdf]

Appendix 2

Google scholar search results (N=23), Included marked Green (N=22)

1. The "Medical Surge Capacity: Workshop Summary" (US institute of Medicine, 2010)
   This paper underscores the critical importance of situational awareness in managing medical surges during public health emergencies. Situational awareness, in this context, refers to the real-time understanding of the evolving crisis, including the number of patients, available resources, and the overall impact on the healthcare system. This awareness is crucial because it:

- **Enables informed decision-making:** Accurate and timely information allows leaders and healthcare providers to make effective decisions regarding resource allocation, patient triage, and overall response strategies.
- **Facilitates resource management:** Situational awareness helps identify resource gaps and enables the efficient deployment of personnel, equipment, and supplies to areas of greatest need.
- **Improves coordination:** A shared understanding of the situation enhances coordination among different agencies, healthcare facilities, and responders, leading to a more cohesive and effective response.
- **Supports timely interventions:** Real-time data allows for the early detection of emerging problems and the implementation of timely interventions to mitigate their impact.
- **Allows for accurate forecasting:** By having a good grasp of the current situation, better predictions of future needs can be made, allowing for better preparedness.

2. **Integrating Disaster Response Tools for Clinical Leadership (Iserson, 2025)**

"Integrating Disaster Response Tools for Clinical Leadership" emphasizes the critical role of situational awareness in effective disaster response, particularly for clinical leaders. The paper discusses how integrating various disaster response tools can significantly enhance this awareness. Key points regarding situational awareness include:

- **Necessity for Real-Time Information:**
  - Clinical leaders require real-time, accurate information to make informed decisions during a disaster. This includes data on patient numbers, injuries, resource availability, and the overall impact of the event.
- **Integration of Tools:**
  - The paper advocates for integrating various tools, such as electronic health records, geographic information systems (GIS), and communication platforms, to create a comprehensive situational picture.
  - This integration allows for the consolidation and analysis of data from multiple sources, providing a clearer understanding of the evolving situation.
- **Enhancing Decision-Making:**
  - Improved situational awareness enables clinical leaders to make timely and effective decisions regarding patient triage, resource allocation, and care delivery.
  - It facilitates the identification of critical needs and the implementation of appropriate response strategies.

- **Facilitating Coordination:**
  - A shared understanding of the situation, facilitated by integrated tools, enhances coordination among different healthcare providers, agencies, and responders.
  - This shared understanding is vital for a unified and effective response.
- **Importance of Data Visualization:**
  - The paper implies that visualizing data through GIS and other methods is important for providing a clear and rapid understanding of the scope and scale of the disaster.
- In essence, the paper focuses on combining data from many sources, to give clinical leaders a complete picture of the disaster, in order to make the best possible decisions.

3. Situation Awareness in multi-agency emergency response (O'Brien, et al. (2020).)

Situational awareness is absolutely crucial in multi-agency emergency response, as it forms the bedrock for effective coordination and decision-making. Here's a breakdown of its importance:

- **Shared Understanding:**
  - In multi-agency responses, numerous organizations (police, fire, medical, etc.) must work together. Situational awareness ensures that all agencies have a shared understanding of the evolving situation, preventing conflicting actions and promoting unified efforts.
- **Effective Coordination:**
  - With accurate and timely information, agencies can coordinate their actions efficiently. This includes resource allocation, task distribution, and communication protocols.
- **Informed Decision-Making:**
  - Leaders from each agency rely on situational awareness to make informed decisions. This includes assessing risks, prioritizing tasks, and adapting strategies as the situation changes.
- **Resource Management:**
  - Situational awareness helps identify resource needs and gaps, enabling agencies to allocate resources effectively. This is particularly important in large-scale emergencies where resources may be scarce.
- **Communication and Interoperability:**
  - Effective communication and interoperability are vital for maintaining situational awareness. This involves sharing information across agencies in a timely and accurate manner.
- **Dynamic Adaptation:**
  - Emergency situations are dynamic and constantly evolving. Situational awareness allows agencies to adapt their response strategies to changing circumstances, ensuring that they remain effective.

In essence, situational awareness in multi-agency emergency response is about creating a common operating picture that enables all stakeholders to work together effectively towards a common goal.

4. Towards Automated Situational Awareness Reporting for ... (Schwarz, et al. (2023)

"Towards Automated Situational Awareness Reporting for Disaster Management—A Case Study" explores the potential of automating situational awareness reporting to improve disaster management. Here's a summary of the key concepts:

- **Problem:**
    - Traditional situational awareness reporting in disaster management is often manual, time-consuming, and prone to errors. This can lead to delays in decision-making and ineffective responses.
- **Solution:**
    - The paper proposes automating the process by integrating various data sources (e.g., sensor networks, social media, satellite imagery) and using data analysis and visualization techniques.
    - The goal is to create real-time, accurate, and comprehensive situational awareness reports that can be easily accessed and understood by decision-makers.
- **Case Study:**
    - The paper presents a case study demonstrating the feasibility and benefits of automated situational awareness reporting.
    - The case study shows how automated systems can collect, process, and present critical information more rapidly and effectively than manual methods.
- **Benefits:**
    - Improved speed and accuracy of situational awareness.
    - Enhanced decision-making capabilities.
    - More efficient resource allocation.
    - Better coordination among responders.
    - Reduced human error.
- **Focus on Automation:**
    - The core of the paper is about how to use computers and data to create a system that automatically generates reports about the current state of a disaster. This allows human responders to concentrate on responding, instead of gathering data.

In short, the paper argues that automated systems can significantly improve disaster management by providing timely and accurate situational awareness reports, enabling faster and more effective responses

5. Technologies Enabling Situational Awareness During ...(Kedia et al., 2020)

"Technologies Enabling Situational Awareness During Disaster Response: A Systematic Review" examines the various technologies that contribute to improved situational awareness in disaster response. Here's a summary of the key findings:

- **Focus on Technology:**
    - The review systematically analyzes existing literature to identify and categorize technologies used to enhance situational awareness in disaster scenarios.
- **Variety of Technologies:**
    - It highlights a wide range of technologies, including:

        - Geographic Information Systems (GIS) for mapping and spatial analysis.
        - Remote sensing (satellite imagery, drones) for real-time monitoring.
        - Sensor networks for detecting environmental changes and hazards.
        - Social media and mobile applications for citizen reporting and information dissemination.
        - Communication technologies (e.g., radio, satellite phones) for reliable communication.
        - Data analytics and AI for processing and visualizing large datasets.
- **Enhancing Key Aspects:**
    - These technologies contribute to:
        - Real-time data collection and analysis.
        - Improved communication and coordination.
        - Enhanced visualization of the disaster situation.
        - Faster decision-making.
        - Better resource allocation.
- **Systematic Review Approach:**
    - The paper utilizes a systematic review methodology, which provides a comprehensive and unbiased overview of the available evidence.
- **Goal:**
    - The goal is to provide a comprehensive overview of how technology is being leveraged to improve situational awareness, and therefore improve disaster response.
- Essentially, this paper is an overview of which modern technologies are currently being used to give responders a better understanding of what is happening during a disaster.

6. "A multi-level analytic framework for disaster situational awareness using Twitter data" (Zhai, 2022) proposes a structured approach to extract valuable situational awareness information from Twitter during disasters. Here's a breakdown:

- **Problem:**
    - Social media, particularly Twitter, is a rich source of real-time information during disasters. However, the sheer volume and unstructured nature of the data make it challenging to extract meaningful insights.
- **Solution:**
    - The paper introduces a multi-level analytic framework to process and analyze Twitter data for disaster situational awareness.
    - This framework breaks down the analysis into multiple levels, allowing for a comprehensive understanding of the situation.
- **Multi-Level Approach:**
    - **Level 1 (Data Collection and Preprocessing):** This level focuses on collecting relevant tweets, filtering out noise, and preparing the data for analysis.

- **Level 2 (Information Extraction):** This level involves extracting key information from the tweets, such as location, event type, and affected areas. This can include Natural language processing (NLP) techniques.
- **Level 3 (Situational Awareness Synthesis):** This level integrates the extracted information to create a comprehensive picture of the disaster situation. This can include visualization and mapping.
- **Level 4(Trend and prediction):** This level uses the prior data to predict future events, and trends in the current event.

- **Twitter Data Utilization:**
    - The framework leverages the real-time nature and widespread use of Twitter to gather information on:
        - Affected areas.
        - Damage assessments.
        - Resource needs.
        - Public sentiment.
- **Goal:** The goal is to provide disaster responders with timely and accurate situational awareness information, enabling them to make informed decisions and respond effectively.
- Essentially, this paper suggests a structured way to use twitter to get a very good understanding of what is happening during a disaster, by processing the data in several stages.

7. Situational-Awareness-in-Emergency-Medicine...... (Lowe et al. 2016)

"Exploring situational awareness in emergency medicine: developing a shared mental model to enhance training and assessment" investigates how to improve situational awareness among emergency medicine professionals. Here's a summary:

- **Focus on Emergency Medicine:**
    - The paper specifically addresses the challenges of maintaining situational awareness in the fast-paced and high-stress environment of emergency medicine.
- **Importance of Shared Mental Models:**
    - It emphasizes the importance of developing shared mental models among team members. A shared mental model means that all members of the team have a similar understanding of the situation, which improves communication and coordination.
- **Enhancing Training and Assessment:**
    - The paper explores how to integrate the concept of situational awareness into emergency medicine training and assessment.
    - This includes developing methods to:
        - Teach trainees how to recognize and interpret critical cues.
        - Assess their ability to maintain situational awareness under pressure.
        - Improve team communication and coordination.
- **Developing a Framework:**

    - The work aims to develop a framework that can be used to improve the understanding and application of situational awareness in emergency medicine practice.
- **Goal:**
    - The overarching goal is to improve patient safety and outcomes by enhancing the situational awareness of emergency medicine professionals.
- Essentially, the paper is about creating a way for emergency room teams to all "be on the same page" so that they can work better together, especially in high stress situations.

8. Just-in-Time Training for Disaster Response in the Austere ...

Just-in-Time Training (JITT) for disaster response in austere environments is a crucial strategy to rapidly equip responders with the necessary skills and knowledge. Here's a summary of its importance and key aspects:

- **Necessity in Austere Environments:**
    - Austere environments, characterized by limited resources and challenging conditions, often require responders to adapt quickly.
    - JITT becomes essential when pre-existing training is insufficient or when novel threats emerge.
- **Focus on Immediate Needs:**
    - JITT delivers training tailored to the specific needs of the current disaster situation.
    - It prioritizes essential skills and knowledge that are immediately applicable to the response effort.
- **Flexibility and Adaptability:**
    - JITT is designed to be flexible and adaptable, allowing responders to learn and apply new skills rapidly.
    - It can be delivered through various methods, including on-site demonstrations, video tutorials, and mobile applications.
- **Addressing Resource Constraints:**
    - In austere environments, resources for traditional training may be limited.
    - JITT provides a cost-effective and efficient way to deliver training without requiring extensive resources.
- **Enhancing Responder Effectiveness:**
    - By providing timely and relevant training, JITT enhances the effectiveness of responders, improving their ability to provide aid and save lives.
- **Key components:**
    - Rapid needs assessment.
    - Development of concise and practical training materials.
    - Delivery of training in the field.
    - Ongoing evaluation and adaptation.

In essence, JITT is about providing the right training, at the right time, and in the right place, to ensure that responders are equipped to handle the challenges of disaster response in resource-limited settings.

9. CRITICALITY OF SUSTAINABLE DISASTER ... (Khairilmizal et al. (2023)

The criticality of sustainable disaster management hinges on the robust interplay of communication, situational awareness, and resource management. Here's a breakdown of why these elements are indispensable:

- **Communication as the Lifeline:**
    - Effective communication is the foundation of any successful disaster response. It ensures that critical information flows between responders, affected populations, and decision-makers.
    - This includes early warnings, real-time updates, and clear instructions.
    - Sustainable disaster management requires establishing resilient communication networks that can withstand the impact of disasters.
- **Situational Awareness as the Compass:**
    - Accurate and timely situational awareness provides a comprehensive understanding of the evolving disaster landscape.
    - It enables responders to assess the extent of damage, identify critical needs, and prioritize actions.
    - Sustainable disaster management relies on continuous monitoring and data analysis to anticipate future needs and adapt response strategies.
- **Resource Management as the Engine:**
    - Efficient resource management ensures that essential supplies, personnel, and equipment are deployed effectively.
    - This includes logistics, procurement, and distribution.
    - Sustainable disaster management emphasizes the responsible use of resources and the development of resilient supply chains.
    - **Interdependence:** These three elements are deeply interconnected. Without good communication, situational awareness is limited. Without good situational awareness, resource management is inefficient. Without good resource management, all other efforts are hampered.
- **Sustainability Implication:**
    - Sustainable disaster management requires building systems that are not only effective during immediate response, but also resilient in the long term. This means investing in infrastructure, training, and community engagement to enhance preparedness and reduce vulnerability.
    - It also means that all three of these pillars must be considered in the pre-disaster stage, and not only when disaster strikes.

In essence, sustainable disaster management is not a one-time event, but an ongoing process that requires continuous improvement in communication, situational awareness, and resource management.

10. **Can Situational Awareness Be Taught? (**De Monnin (2024).

Yes, situational awareness can definitely be taught, although it's a complex skill that requires a combination of knowledge, practice, and experience. It's not simply an innate ability. Here's how it's approached:

Key Components of Teaching Situational Awareness:

- **Knowledge Acquisition:**

    - Providing individuals with the foundational knowledge they need to understand their environment and potential threats. This includes:
        - Understanding relevant procedures and protocols.
        - Recognizing critical cues and indicators.
        - Learning about potential hazards and risks.
- **Skill Development:**
    - Training individuals to develop the cognitive skills necessary for situational awareness, such as:
        - Active observation and monitoring.
        - Information processing and analysis.
        - Anticipation and prediction.
        - Decision-making under pressure.
- **Practice and Simulation:**
    - Providing opportunities for individuals to practice their situational awareness skills in realistic scenarios. This can include:
        - Simulations and role-playing exercises.
        - Scenario-based training.
        - Real-world exercises and drills.
- **Feedback and Debriefing:**
    - Providing constructive feedback and debriefing after training exercises and real-world events. This helps individuals to:
        - Identify areas for improvement.
        - Learn from their mistakes.
        - Reinforce positive behaviors.
- **Developing Mental Models:**
    - Training people to build accurate and adaptable mental models of their operational environment. This involves:
        - Understanding the relationships between different elements.
        - Developing a sense of "what should be happening."
        - Recognizing deviations from the expected.
- **Promoting Teamwork and Communication:**
    - In many situations, situational awareness is a team effort. Training should emphasize:
        - Effective communication and information sharing.
        - Coordination and collaboration.
        - The development of shared mental models.

Methods used to teach it:

- Classroom lectures, and instruction.
- Simulation, from simple table top exercises, to complex virtual reality simulations.
- On the job training, with experienced personnel mentoring less experienced.
- Case studies, and after action reviews.

While some individuals may have a natural aptitude for situational awareness, effective training can significantly improve the skills of anyone.

11. Non-technical skills needed by medical disaster responders (Westman et al, 2024)

A scoping review of non-technical skills for medical disaster responders highlights that beyond medical expertise, a range of interpersonal and cognitive abilities are crucial for effective performance. Here's a summary of the key findings:

- Communication:
  - Clear and concise communication is vital for coordinating with other responders, providing instructions to victims, and conveying critical information under stress.
  - This includes active listening, cross-cultural communication, and the ability to communicate effectively in chaotic environments.
- Teamwork and Leadership:
  - Disaster response often involves working in multidisciplinary teams, requiring strong teamwork and collaboration skills.
  - Leadership skills are essential for coordinating efforts, making decisions under pressure, and motivating team members.
- Decision-Making and Problem-Solving:
  - Responders must be able to make rapid and effective decisions in dynamic and uncertain situations.
  - This includes the ability to prioritize tasks, adapt to changing circumstances, and solve complex problems with limited resources.
- Situational Awareness:
  - Maintaining a clear understanding of the evolving situation is critical for effective response.
  - This includes the ability to gather and process information, anticipate potential hazards, and recognize changes in the environment.
- Stress Management and Resilience:
  - Disaster response can be emotionally and physically demanding, requiring responders to manage stress and maintain resilience.
  - This includes the ability to cope with trauma, manage fatigue, and maintain a positive attitude.
- Ethical Reasoning:
  - Disaster situations can present difficult ethical dilemas, requiring responders to make sound ethical judgements, often with limited information.
- Flexibility and Adaptability:

    - Disaster response is inherently unpredictable. Responders must be able to adapt to changing conditions, and be flexible in their approach.
- Cultural Competence:
    - Disasters can affect diverse populations, making cultural competence essential for providing effective and sensitive care.

In essence, medical disaster responders need a well-rounded skillset that goes beyond clinical knowledge. These non-technical skills are essential for ensuring effective coordination, communication, and decision-making in the challenging and stressful environment of disaster

12. Development of Competencies for Disaster Medicine ... (Am Coll Emerg Phys, 2014)

The document "Development of Competencies for Disaster Medicine Fellowships" emphasizes the need for specialized training in disaster medicine, and within that, highlights key competencies including situational awareness and a "disaster mindset." Here's a summary focused on those aspects:

- **Situational Awareness as a Core Competency:**
    - The document recognizes situational awareness as a critical skill for disaster medicine fellows.
    - It implies that fellows must be able to rapidly assess and interpret complex and dynamic disaster environments.
    - This includes gathering and analyzing information from various sources, identifying potential hazards, and anticipating future needs.
    - The ability to maintain situational awareness is crucial for effective decision-making and resource allocation during disasters.
- **Cultivating a "Disaster Mindset":**
    - The document underscores the importance of developing a "disaster mindset," which goes beyond traditional clinical thinking.
    - This mindset involves:
        - Thinking at a population level, rather than just individual patient care.
        - Understanding the unique challenges of resource-constrained environments.
        - Being prepared to operate in chaotic and unpredictable situations.
        - Being able to adapt to changing circumstances.
        - Being able to make ethical decisions under high stress and with limited resources.
    - The "disaster mindset" also includes the ability to anticipate and prepare for the psychological and social impacts of disasters on both victims and responders.
- **Competency-Based Training:**
    - The document promotes a competency-based approach to disaster medicine fellowship training, ensuring that fellows acquire the necessary skills and knowledge to effectively respond to disasters.
    - This means that training is designed to produce measurable skills, rather than just theoretical knowledge.
- **Integration:**

- The document implies that situational awareness and the disaster mindset are not isolated skills, but rather integrated components of a broader set of competencies needed for effective disaster response.

In essence, this document stresses that disaster medicine fellowships must train individuals to not only have the medical skills, but the ability to rapidly understand chaotic situations, and to think in a way that is effective during a disaster.

13. "An Emergency Response Model toward Situational Awareness Improvement" (Sapateiro and Antunes (2009). presents a structured framework designed to enhance situational awareness during emergency responses. Here's a summary of its core components:

- **Focus on Improvement:**
  - The primary goal of the model is to improve the quality and effectiveness of situational awareness in emergency scenarios.
- **Structured Framework:**
  - It proposes a model that organizes the process of gathering, processing, and disseminating information to improve understanding of the situation.
- **Key Elements Likely Included:**
  - **Data Collection:**
    - The model would emphasize the importance of gathering data from diverse sources, including sensor networks, communication systems, and human observers.
  - **Information Processing:**
    - It would outline methods for analyzing and interpreting the collected data, transforming it into meaningful information.
  - **Information Dissemination:**
    - The model would address how to effectively communicate the processed information to relevant stakeholders, including responders and decision-makers.
  - **Decision Support:**
    - It likely includes methods to aid in decision making based on the processed information.
  - **Feedback Loops:**
    - It will likely include ways to improve the process, by using information gathered during the response.
- **Situational Awareness as a Process:**
  - The model likely views situational awareness as a dynamic and iterative process, requiring continuous monitoring and adaptation.
- **Goal:**
  - The overarching aim is to enable responders to make more informed and timely decisions, leading to more effective emergency responses.

In essence, this model provides a systematic approach to improve how emergency responders understand what is happening during an event, so that they can respond more effectively.

14. Lateef F (2022) Tying in Situational Awareness, Clinical

This concept proposes a powerful approach to enhancing emergency medicine practice by integrating situational awareness, clinical reasoning, and clinical judgment through cross-training using case-based discussions. Here's a breakdown:

- **Integration of Core Skills:**
    - The approach recognizes that effective emergency medicine relies on the interplay of three crucial skills:
        - **Situational Awareness:** Understanding the patient's condition, the environment, and potential risks.
        - **Clinical Reasoning:** Analyzing information, formulating hypotheses, and developing a differential diagnosis.
        - **Clinical Judgment:** Making informed decisions about patient care, based on available evidence and clinical expertise.
- **Cross-Training with Case-Based Discussions:**
    - The proposed method utilizes cross-training, where professionals from different backgrounds or specialties collaborate to discuss real or simulated patient cases.
    - Case-based discussions provide a platform for:
        - Sharing perspectives and insights.
        - Exploring different approaches to diagnosis and treatment.
        - Analyzing the factors that influence situational awareness, clinical reasoning, and clinical judgment.
        - Practicing the skills in a controlled environment.
- **Benefits:**
    - **Enhanced Situational Awareness:** By discussing cases, participants can learn to recognize subtle cues and develop a more comprehensive understanding of complex situations.

15. Situation awareness in emergency medicine (Levin and Sauer, 2012)

Situational awareness (SA) is a critical cognitive skill in emergency medicine, playing a vital role in patient safety and effective care. In the fast-paced and high-stakes environment of the emergency department (ED), maintaining accurate and timely SA is essential for making informed decisions. Here's a breakdown of its significance:

Key Aspects of Situational Awareness in Emergency Medicine:

- **Understanding the Patient's Condition:**
    - This involves gathering and interpreting information about the patient's medical history, presenting symptoms, and vital signs.
    - It requires the ability to recognize subtle cues and patterns that may indicate underlying medical conditions.
- **Assessing the Environment:**

    - This includes understanding the layout of the ED, the availability of resources, and the presence of potential hazards.
    - It also involves being aware of the overall patient flow and the workload of the team.
- **Anticipating Future Events:**
    - This involves predicting potential complications and developing contingency plans.
    - It requires the ability to recognize early warning signs of deterioration and to anticipate the need for interventions.
- **Decision-Making Under Pressure:**
    - SA provides the foundation for making rapid and effective decisions in time-critical situations.
    - It enables clinicians to prioritize tasks, allocate resources, and coordinate care effectively.
- **Team Situational Awareness:**
    - In the ED, SA is often a team effort.
    - Effective communication and information sharing are essential for developing a shared understanding of the situation.
    - This includes the ability to recognize and address discrepancies in team members' SA.
- **Challenges:**
    - The ED environment is characterized by high workload, interruptions, and time pressure, which can impair SA.
    - Fatigue, stress, and distractions can also contribute to errors in SA.
- **Improving SA:**
    - Training programs can help clinicians develop and maintain SA skills.
    - Simulation exercises and case-based discussions can provide opportunities to practice SA in realistic scenarios.
    - Checklists and cognitive aids can also help to improve SA.

In essence, situational awareness in emergency medicine is about being constantly aware of the patient's condition, the surrounding environment, and potential future events, to provide the best possible care.

16. Case Study of COVID-19 | SN Computer Science (Bouzidi et al., 2022)

This study, focused on the COVID-19 pandemic, highlights the critical need to enhance warning systems, situational awareness, assessment capabilities, and public education in managing emergencies. Here's a summary:

- **COVID-19 as a Case Study:**
    - The COVID-19 pandemic served as a stark reminder of the vulnerabilities in existing emergency management systems.
    - It demonstrated the need for rapid and effective responses to novel and evolving threats.
- **Enhancing Warning Systems:**
    - The study emphasizes the importance of early warning systems that can detect and track emerging threats.

    - This includes strengthening surveillance systems, improving data sharing, and developing predictive models.
    - It also means that warnings must be delivered in a timely, and effective manner, to the correct audience.
- **Improving Situational Awareness:**
    - The pandemic highlighted the need for accurate and timely situational awareness.
    - This involves gathering and analyzing data from diverse sources, including public health surveillance, social media, and news reports.
    - It also requires the ability to communicate this information effectively to decision-makers and the public.
- **Strengthening Assessment Capabilities:**
    - The study underscores the importance of rapid and accurate assessment of the impact of emergencies.
    - This includes assessing the spread of disease, the capacity of healthcare systems, and the economic and social consequences.
    - It also means having the ability to rapidly develop, and deploy, new testing methods.
- **Enhancing Public Education:**
    - Effective public education is crucial for promoting compliance with public health measures and mitigating the spread of disease.
    - This includes communicating accurate information about the risks, providing guidance on preventive measures, and addressing misinformation.
    - This also means that education needs to be tailored to the specific needs of different populations.
- **Lessons Learned:**
    - The COVID-19 pandemic provided valuable lessons for improving emergency management systems.
    - By enhancing warning systems, situational awareness, assessment capabilities, and public education, we can better prepare for and respond to future emergencies.

In essence, this study uses the COVID-19 pandemic to illustrate the importance of a holistic approach to emergency management, emphasizing the interconnectedness of warning, situational awareness, assessment, and education.

17. AI-Enabled Situational Awareness in Disaster Response

https://www.jhuapl.edu › sites › default › files › A...

av JD Lambert (2021)— **Situational awareness during disaster response** is critical as it enables the response community to rapidly and efficiently assist those in urgent need ...

Why COVID-19 Situational Awareness Still Matters as ...

https://www.haponline.org › wh...

28 feb. 2023 — **Situational awareness** does not have to stay in the lane of one person or department. **Emergency** managers, infection preventionists, employee ...

18. A New Mindset for Emergency Response (Government tech, 2022)

e.Republic

https://media.erepublic.com › document › GT22_...

first responders' **situational awareness**, put relevant data at their ... health clinics or new family health **programs**, these technologies can help ...

19. An Upstream-Downstream Approach for Disaster ... (Kuziemsky et al. 2o12)

ISCRAM Digital Library

https://idl.iscram.org › 24_Kuziemsky_etal2012

av CE Kuziemsky · Citerat av 13 — Our objective was to develop a system to support the development of **situational awareness** about functional capacities and appropriate supports, and to ...

20. A protocol for analysing the role of shared situational ...

Aalto-yliopisto

https://research.aalto.fi › files › 1_s2.0_S2212420...

av M Laurila-Pant · 2023 · Citerat av 21 — **situational awareness** and decision-making in cooperative **disaster** simulations. International Journal of **Disaster**. Risk Reduction, 86, Article ...

21. Measuring situation awareness in emergency settings (cooper et al, 2013)

"Measuring situation awareness in emergency settings: a systematic review of tools and outcomes" systematically examines the various tools and methods used to measure situational awareness (SA) in emergency contexts, and explores the outcomes associated with these measurements. Here's a summary:

- **Focus on Measurement:**
    - The review addresses the critical need to quantify and assess SA in emergency environments, acknowledging its importance for effective decision-making and performance.
- **Systematic Review Approach:**
    - It employs a systematic review methodology, ensuring a comprehensive and unbiased analysis of existing research.
- **Tools and Methods:**
    - The review identifies and categorizes the various tools and methods used to measure SA, which may include:
        - Subjective measures (e.g., questionnaires, self-assessments).
        - Objective measures (e.g., performance metrics, real-time probes).
        - Simulation-based assessments.
        - Physiological measures.
    - It likely evaluates the strengths and limitations of each approach.
- **Emergency Settings:**

- The review focuses specifically on emergency settings, such as:
  - Emergency medical services (EMS).
  - Emergency departments (EDs).
  - Disaster response.
  - Firefighting.
- **Outcomes:**
  - The review explores the outcomes associated with SA measurements, such as:
    - Performance effectiveness.
    - Decision-making accuracy.
    - Team coordination.
    - Patient safety.
    - Training effectiveness.
- **Goal:**
  - The overarching goal is to provide a comprehensive overview of the current state of SA measurement in emergency settings, and to identify areas for future research and development.
- **Practical Application:**
  - The findings of the review can inform the selection and implementation of SA measurement tools in emergency settings, leading to improved training, performance, and patient outcomes.

In essence, this review provides a valuable resource for researchers and practitioners seeking to understand and measure situational awareness in the challenging context of emergency response.

22. Immersive Simulation and Paramedicine Students ... (Williams et al, 2022)

This pilot study investigates the impact of immersive simulation on paramedicine students' situational awareness, using a mixed-methods approach (combining quantitative and qualitative data). Here's a summary:

- **Focus on Paramedicine Students:**
  - The study targets paramedicine students, who require strong situational awareness skills in their demanding profession.
- **Immersive Simulation:**
  - Immersive simulation, likely involving virtual reality or high-fidelity scenarios, is used as an educational intervention.
  - This method aims to create realistic and engaging learning experiences that replicate the challenges of real-world emergencies.
- **Situational Awareness Assessment:**
  - The study measures students' situational awareness using both quantitative and qualitative methods.
  - Quantitative methods may include standardized tests or performance metrics.
  - Qualitative methods likely involve interviews, observations, or surveys to gather insights into students' perceptions and experiences.

- **Mixed-Methods Approach:**
    - The use of a mixed-methods approach allows researchers to gain a more comprehensive understanding of the impact of immersive simulation.
    - Quantitative data provides objective measures of performance, while qualitative data provides rich insights into students' cognitive processes and experiences.
- **Pilot Study:**
    - As a pilot study, this research is designed to explore the feasibility and effectiveness of the intervention and to inform future, larger-scale studies.
    - Pilot studies are used to test and refine research methods before they are used in larger, more expensive studies.
- **Goal:**
    - The goal is to determine if immersive simulation can effectively enhance paramedicine students' situational awareness.
    - Ultimately, this research aims to improve the training of paramedics and enhance patient safety in emergency situations.

In short, this research is testing if using very realistic simulations can improve how well Paramedic students are able to understand and react to emergency situations.

23. Health and Safety Situation Awareness Model ...(GU et al, 2019)

This title indicates a research or development project focused on creating a comprehensive health and safety situational awareness model for emergency management, utilizing multi-sensor signal fusion. Here's a breakdown of the key concepts:

- **Health and Safety Situation Awareness Model:**
    - This implies the development of a structured framework to understand and track the real-time health and safety conditions within an emergency scenario.
    - It would encompass factors like:
        - Environmental hazards (e.g., toxic gases, radiation, structural instability).
        - Patient health status (e.g., vital signs, injuries, exposure levels).
        - Responder safety (e.g., location, exposure, fatigue).
    - The goal is to provide a holistic and up-to-date picture of the situation.
- **Emergency Management:**
    - This model is designed to support emergency response efforts by providing critical information for decision-making.
    - It aims to enhance:
        - Incident command and control.
        - Resource allocation.
        - Responder safety.
        - Victim triage and treatment.

- **Multi-Sensor Signal Fusion:**
    - This is a crucial aspect of the model, involving the integration of data from various sensors.
    - Potential sensor types include:
        - Environmental sensors (e.g., gas detectors, radiation monitors, temperature sensors).
        - Wearable sensors (e.g., vital sign monitors, GPS trackers).
        - Imaging sensors (e.g., thermal cameras, drones).
    - Signal fusion combines the data from these diverse sources to create a more accurate and reliable representation of the situation.
    - This fusion of data combats the potential failure of a single sensor, and also provides a more complete data set.
- **Benefits:**
    - Improved accuracy and reliability of situational awareness.
    - Enhanced decision-making capabilities.
    - Increased responder safety.
    - More effective victim care.
    - Faster response times.

In essence, this project aims to create a technological solution that combines data from many sources, to give emergency responders a very clear, and accurate picture of what is happening, so they can make the best possible decisions.
